# "Just Another Tool for Online Studies” (JATOS): An Easy Solution for Setup and Management of Web Servers Supporting Online Studies

**DOI:** 10.1371/journal.pone.0130834

**Published:** 2015-06-26

**Authors:** Kristian Lange, Simone Kühn, Elisa Filevich

**Affiliations:** Department of Lifespan Psychology, Max Planck Institute for Human Development, Berlin, Germany; Max Planck Institute for Human Cognitive and Brain Sciences, GERMANY

## Abstract

We present here “Just Another Tool for Online Studies” (JATOS): an open source, cross-platform web application with a graphical user interface (GUI) that greatly simplifies setting up and communicating with a web server to host online studies that are written in JavaScript. JATOS is easy to install in all three major platforms (Microsoft Windows, Mac OS X, and Linux), and seamlessly pairs with a database for secure data storage. It can be installed on a server or locally, allowing researchers to try the application and feasibility of their studies within a browser environment, before engaging in setting up a server. All communication with the JATOS server takes place via a GUI (with no need to use a command line interface), making JATOS an especially accessible tool for researchers without a strong IT background. We describe JATOS’ main features and implementation and provide a detailed tutorial along with example studies to help interested researchers to set up their online studies. JATOS can be found under the Internet address: www.jatos.org.

## Introduction

### Running studies online

Online data collection is a promising alternative in many fields of research. Although online data collection has some shortcomings [1,2], it remains a viable alternative to experiments conducted in laboratories. It is especially useful in cases where laboratory-based data collection is too expensive or simply not feasible, like studies of particularly large or diverse populations [3] or of micro-longitudinal data [1,4].

Online studies have proven robust and informative even in cases where traditional approaches are also viable. Results from online data replicate those obtained through telephone interviews [5]. Crump *et al*. [6] reliably reproduced several classic results from behavioral psychology, including for example the Stoop task [7], the Go/No-Go task [8] and the attentional blink task [9]. Further, Barnhoorn *et al*. [10] developed tools that provide precise timing control in a browser environment, with which they replicated the positive- and negative compatibility effects typically found in a masked priming task [11].

### Existing tools for online studies

In order to make studies available to anybody with Internet access, these should be hosted (saved) on a server. A server, in short, is a computer that is accessible through the Internet.

Multiple tools are readily available for hosting online studies. Three of the most widely used tools include the two commercial platforms Amazon Mechanical Turk (AMT, https://www.mturk.com/) and Qualtrics (http://www.qualtrics.com/), and the open source psiTurk (https://psiturk.org/). Both AMT and Qualtrics can help to set up a survey’s *client-side*, which is what participants see in their browsers (see the Glossary for a clarification of the technical terms used in this paper). These platforms then store the survey data in their own dedicated servers. psiTurk, in turn, does not help with the client-side, but instead helps researchers to set up their own *server-side*, and store their resulting data privately. AMT mainly serves as a marketplace to recruit and pay study participants, but researchers can also compose basic surveys via AMT’s graphical user interface (GUI). Qualtrics greatly extends AMT’s survey functionality, and allows researchers to assemble potentially very elaborate surveys using virtually only its GUI, and thus without needing to write any scripts. Qualtrics surveys can be connected to AMT, but can also be run independently.

The number of users of both AMT and Qualtrics attest to their potential and versatility: currently each of these platforms hosts an average of approximately 250.000 projects at any given point in time. They both have, however, two important limitations. First, researchers can only build surveys by modifying predefined templates. This limits the options available for study scripts, although, importantly, new tools are emerging that provide some of the functionality missing in the basic templates-most notably, QRTEngine provides precise timing control for Qualtrics-based JavaScripts [10]. Second, and importantly, all data are stored in either AMT’s or Qualtrics’ dedicated servers. This clashes with the data privacy policies of most research institutes, which require that data are stored securely in their own local servers.

PsiTurk allows researchers to set up and control their own web servers. In this way, psiTurk servers meet some important data protection requirements. Additionally, unlike AMT or Qualtrics servers, psiTurk servers will run virtually any custom study script; allowing researchers to make use of the many JavaScript libraries available to present stimuli, collect responses or control timing. While psiTurk is a very powerful platform, it runs exclusively on Unix-like operating systems (*i*.*e*., Linux and Mac OS X, but not in Microsoft Windows). In addition, researchers must communicate with psiTurk via the command-line interface. This makes psiTurk less accessible to non-tech-savvy researchers who might not be familiar with the command line.

Finally, a number of libraries are available that help to write the scripts that run on the client-side. For example, jsPsych (http://www.jspsych.org/) is a JavaScript library tailored for psychology experiments [12]. QRTEngine, mentioned above, enables tight timing control in JavaScript-based Qualtrics surveys; whereas ScriptingRT [13] provides the tools for tight timing measurement and control in studies written in Flash.

### JATOS: a new tool for online studies

We developed “Just Another Tool for Online Studies” (JATOS) as an alternative for researchers who are not comfortable with the command line, and more generally to any researcher that wants to run custom online experiments. JATOS is an open source, cross-platform web application to set up and run studies online. Two main features make JATOS especially accessible. First, its GUI allows non tech-savvy researchers to create, modify, and manage their studies and results. Second, it can be quickly installed on either a local computer or on a server. The local installation is straightforward as it requires nothing more than a computer running Java (https://www.java.com/) and a browser. It allows researchers to write and try their study scripts locally and privately before setting up full-fledged web server, accessible via the Internet. The installation on a server is equally straightforward, although some standard knowledge of Internet technologies and security is required. However, once installed, JATOS is entirely GUI-based and does not require any server knowledge or even the command line. Thus, JATOS’ second

Like psiTurk, JATOS does not aim to help researchers write their study scripts. Instead, it provides a helpful framework to run custom scripts; it manages study participants, preventing repeated participation, and it allows researchers to easily access and manage study results through a GUI. Like psiTurk, then, running a study in JATOS does require researchers to write their own scripts (in JavaScript) for their studies. Importantly, JavaScript is easy to learn. It is the most widely used language across the Internet and extensive community support is available, along with uncountable open source libraries and helpful resources. In addition, we provide example study scripts that researchers can modify and use as guides to write their own.

JATOS and its Wiki pages are available under the following link: http://www.jatos.org/

### Compatibility with existing tools

Running a study online requires solutions at multiple levels; and different tools cater to different needs: they may provide means to recruit participants, client-side solutions, or server-side settings. Ideally then, individual tools should be compatible with each other. JATOS focuses on server-side settings, and is thus compatible with some of the currently available tools that provide solutions for other aspects of online testing. For example, JATOS and jsPsych are complementary: the former helps to set up the server-side and the latter helps to write client-side scripts. In addition, JATOS is compatible with AMT and can be used to run what AMT dubs “external human intelligence tasks” (external HITs) in a straightforward way. JATOS also detects whether participants accessed a study via AMT or not, and generates the confirmation codes necessary to confirm participation and payment through AMT.

JATOS is at the moment not compatible with other available tools for online studies. For example, JATOS is not compatible with ScriptingRT, which supports Flash-based studies running on the client-side. Unlike with the native JavaScript, a browser plug-in is necessary to run studies written in Flash. For this reason, we prioritized JavaScript in JATOS, and at the moment do not support studies written in Flash. Likewise, JATOS is not compatible with QRTEngine, as it has some Qualtrics dependencies and therefore does not work as a standalone library. QRTEngine however, is a proof of concept that tight timing control *is* possible in a browser and suggests that a stand-alone tool could be developed soon.

JATOS is incompatible with other tools with overlapping purposes, such as PsiTurk, WebExp [14] or Tatool [15]. These three tools provide different server-side solutions, along with different degrees of client-side support. While the choice of tool is somewhat a matter of taste, we believe that JATOS provides the most accessible and lightweight alternative, and at the same time the most intuitive GUI to write, run and manage online studies.

### So what does JATOS actually do?

In short, if a researcher wishes to set up her own web server and conduct studies online, she would need to: set up an HTTP server (e.g. Apache or Nynx), set up a database and communicate with it (e.g. via SQL commands) and programmatically manage permissions to handle participants’ access. JATOS provides an out-of-the-box solution for all of the above, and a GUI for further communication with the server and database.

In what follows we give an overview of JATOS’ main features and then describe its implementation, to encourage open-source collaboration. We end with a detailed tutorial and some recommendations, drawn from our own experience, to help interested researchers make the best use of JATOS and maximize its versatility. We also clarify the terminology that we use to refer to JATOS in a glossary.

## Overview of JATOS

### 1. General features

#### 1.1. Cross-platform compatibility and easy installation

JATOS is free to use and open source, released under the Apache 2 license. The complete source code is freely available in GitHub (https://github.com/JATOS/JATOS). It runs on all three common platforms (Microsoft Windows, Mac OS X, and Linux), both in the local and server instances. The more straightforward local instance runs on a local computer and is not accessible through the Internet. It is therefore not enough to run an online study. Its main advantage is that it is extremely quick to set up, and it does not require an active server or even an Internet connection. This allows individual researchers to write their experiments, before engaging in the more complex task of setting up a server. Installing JATOS on a server is as straightforward as installing it on a local computer, but has some additional requirements, like an IP address and port (see “Download and install JATOS on a server” in the Tutorial section for alternatives to make a simple local installation accessible through the Internet).

#### 1.2. Intuitive GUI

**Fig 1:**
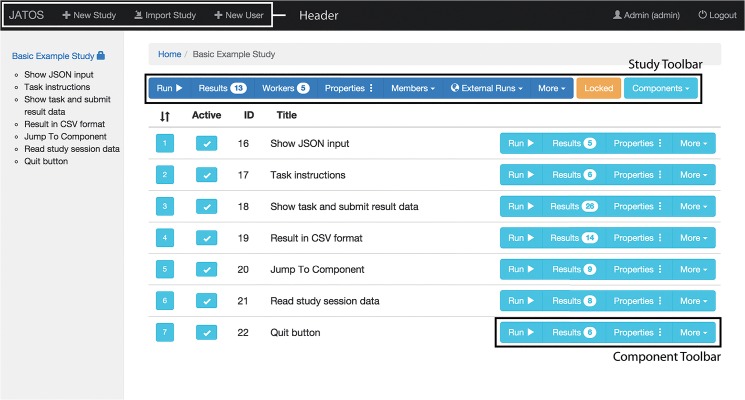
Screenshot of JATOS’ GUI. An example study page, showing the “Basic example study” (available for download from the Wiki pages). The header is the black bar on the top of the screen, the study toolbar is dark blue and each component’s toolbar is light blue.

The GUI is one of JATOS’ main strengths (see Fig 1). All communication with the JATOS server can be done through the GUI, including access to and management of result data. This completely eliminates the need for the command line interface, which is often the standard way to communicate with a server and database. As a result, testing and data analysis are much more accessible to researchers not necessarily familiar with the command line.

#### 1.3. User management

Multiple users can access a single JATOS instance through individual, password-protected accounts. Once a user has created a study, she can give other users editing privileges for it (namely, including them as study **members**). Users can only see and edit those studies that include them as members.

#### 1.4. Worker management

Different people (workers) run a study for different purposes. Researchers will typically test a study or any of its individual components by running it internally (*i*.*e*., within JATOS, by clicking on the ‘Run’ buttons). Workers that access the study internally are assumed to be testers, and are therefore allowed to run the same study as many times as they wish. It is also of course possible to run a study externally, (*i*.*e*., from outside JATOS, by following a direct link to the study). JATOS supports and manages different types of external links and workers, and can be set to allow some workers repeated access to the same study, while making sure that others access it only once. Different worker types can be simultaneously enabled. See the Wiki pages for full details on the supported worker types.

#### 1.5. Embedded H2 database, optional MySQL database

JATOS is by default paired with an H2 database (http://www.h2database.com/). Compared with other more widely used databases like MySQL (http://www.mysql.com/), the H2 database can be embedded into the application and thus requires no additional setup. Since it is a relatively new technology some researchers might be concerned with data stability and opt for the more widely used MySQL database. JATOS was extensively tested with both H2 and MySQL databases. See the Wiki pages for details and installation instructions.

#### 1.6. Run multiple studies in parallel

A single JATOS instance can handle multiple studies. These studies can be edited simultaneously by multiple users and run simultaneously by multiple workers.

### 2. Security, ethics and data privacy standards

#### 2.1. Data avoidance and minimisation

Based on the principle of data avoidance and data minimisation, JATOS stores data only where necessary or explicitly told to do so by the researcher. JATOS stores neither system nor browser information, nor IP addresses. Study results are stored together with a numerical worker ID without any further personal information. If AMT workers are recruited for a study, the AMT worker ID is additionally stored. This is necessary to allow researchers to confirm payment to participants. JATOS does not restrict what information is stored, and researchers can of course store personal or private data, for which they are responsible.

#### 2.2 Abort study method

As in any laboratory-based study, workers should be allowed to quit a study at any time. The easiest way to do this is to close the browser window, but in this case their result data would remain in the server’s database. In agreement with ethics requirements, jatos.js provides a function (jatos.abortStudy) that will be triggered if workers (e.g.) click on a “Quit study” button that researchers included in their study’s scripts. This function deletes all the participant’s result data (but not the metadata) from the database, without intervention from a researcher.

### 3. Study setup

#### 3.1. Import/export of studies and components

JATOS can easily export and import entire studies, or individual components. This serves two purposes. First, it enables a quick and hassle-free startup, as researchers can import example studies and explore JATOS right away. Second, it encourages collaborations and replications, by facilitating the exchange of study scripts. Studies are exported as.zip files that include all the study and component scripts, assets, JSON input and properties, but exclude all result data.

#### 3.2. Flexible study flow

JATOS encourages, but does not enforce, studies composed of multiple components. If a study contains multiple components and is conveniently designed, single clicks can quickly change the entire study structure. Individual components can be toggled between the active and inactive states. Inactive components are completely ignored from the study flow, effectively removing big parts of code from what is displayed on the client-side. Importantly, two alternatives are available for the transition between components. First, a study can follow a strictly linear flow, in which components follow one another in the client-side, matching their order in the server-side. Alternatively, non-linear study flows are possible: JATOS can jump to any given component and thus enables loops, repetitions, and component exclusions, based on participants’ responses.

#### 3.3. Change JSON input to change study parameters

Each component’s JSON input can be set to specify study parameters that modify a task. For example, a word memory task can be made more difficult by increasing the number of trials, changing the words to be remembered, reducing presentation times or increasing the delay to memory recall. Researchers can write their scripts to read the JSON input and take from it the values of a component’s parameters. Then, these parameters can be easily changed via the GUI without the need to modify the scripts themselves. This approach is completely optional, since all study parameters can also be hard-coded into a component’s script. If used wisely, however, this option can add a great deal of versatility to studies and make them more accessible for researchers that prefer to avoid writing code in (the sometimes confusing) JavaScript.

### 4. Result data management and export

#### 4.1. Result data management

Researchers can inspect, delete, and export result data from within JATOS’ GUI, using interactive table views that include filtering and sorting options.

#### 4.2 Result data export

JATOS exports result data in a simple text file that can later be opened with any text editor or data analysis program. JATOS does not enforce any output data format, other than the requirement that it is text-based. Therefore it naturally supports the widely used data formats JSON, XML and comma-separated values (CSV).

## JATOS’ Design and Implementation

JATOS is written in Java and uses the Play Framework (https://www.playframework.com), a light-weight web application framework that runs on a Java Virtual Machine. JATOS (as any other Play-based application) incorporates three main features. First, it includes an embedded Netty web server (http://netty.io/). As a result, it is not necessary to set up an external web server like Apache or Nginx. Second, JATOS is stateless (also known as RESTless). Third, it uses Akka (http://akka.io/) for concurrency and fault tolerance, making it stable and robust.

### Architecture

**Fig 2:**
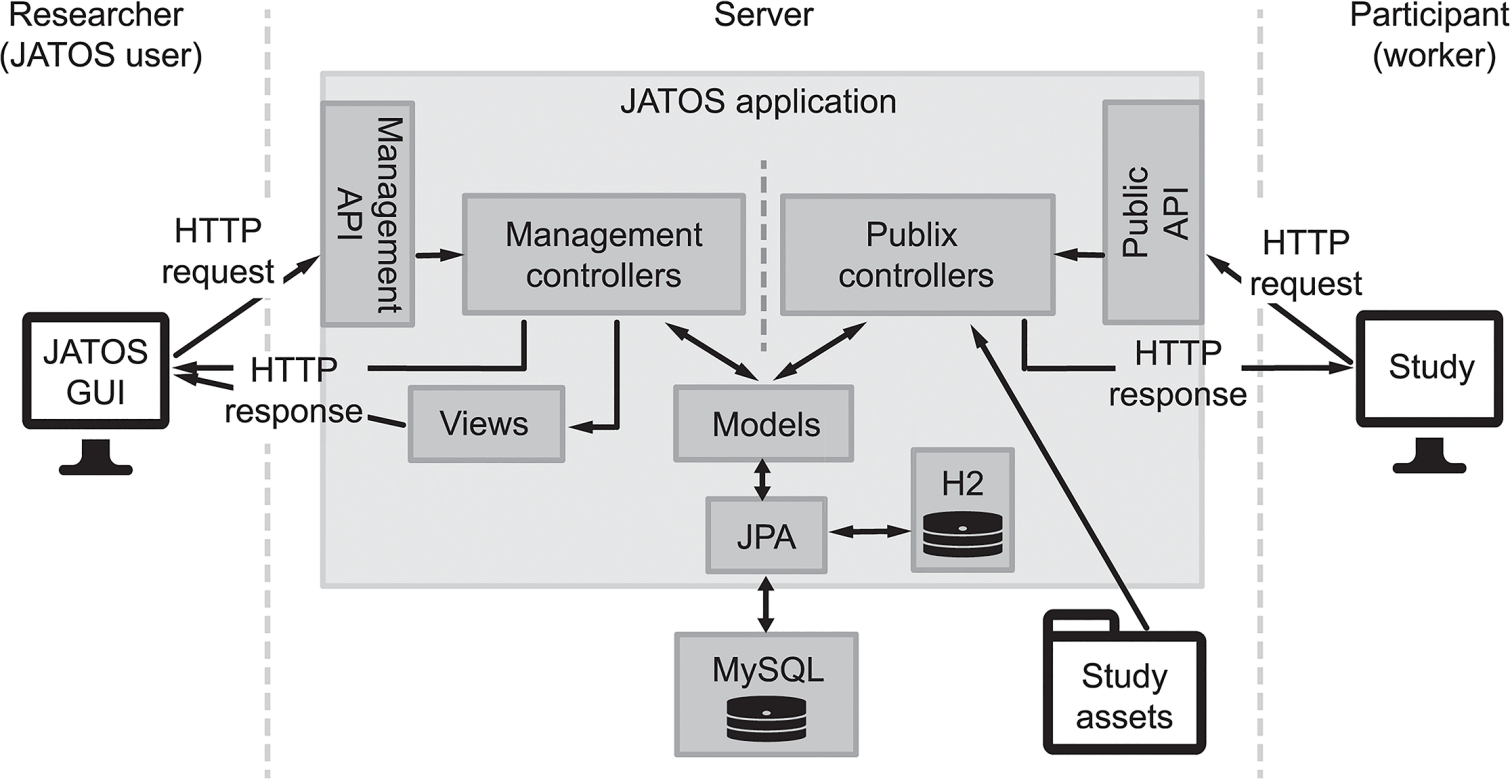
JATOS’ architecture. JATOS follows the MVC architecture. See Implementation section for details.

JATOS follows the model-view-controller (MVC) architecture (see Fig 2). Incoming HTTP requests are routed to controllers that in turn return an HTTP response. Controllers fall into two groups. On the one hand, the *management* controllers answer calls sent from JATOS’ GUI, via the *management* API. On the other hand, the *Publix* controllers handle requests originating from the participant’s browser and going via the public API. If the browser asks to start a component, the Publix controller retrieves the HTML file associated with the component from the study assets and returns it.

### Database

JATOS uses Hibernate (http://hibernate.org/), an implementation of the Java Persistence API (JPA). This separates access to the relational database from the rest of the application and databases can thus be easily exchanged. In particular, JATOS was thoroughly tested with H2 and MySQL.

## Jatos Tutorial

We provide a detailed tutorial that covers most of JATOS’ functionality. Up-to-date instructions can also be found in the JATOS Wiki. If you have any questions, you can post them, or find answers, in the mailing list (jatos@googlegroups.com). We will update JATOS (and especially the jatos.js library) periodically, so we recommend that you check JATOS’ webpage for significant updates.

### 1. Startup

#### Step 1.1: Download JATOS

Download the latest release from the GitHub link: https://github.com/JATOS/JATOS/releases.

#### Step 1.2: Install and launch JATOS locally

We recommend that you write and debug all studies in a local instance first. The main benefit of this is that all study assets are stored in the local computer, and are therefore easily accessible through each system’s file manager. A server’s file system can be more difficult to navigate because a file manager is often not available. Working on a local instance, then, helps you identify and handle script errors, name conflicts, etc. Once you are done writing and debugging your study locally, you can confidently upload all assets to the server instance: simply export a given study through the local GUI and import it into the server through the server GUI (see Step 2.9 in this tutorial).

Installation instructions depend on your operating system and can be found in the JATOS Wiki.

Once you followed the platform-specific installation instructions, go to your browser of choice and type http://localhost:9000/ in the address bar. Wait a few moments if the page fails to load, and refresh the page. Log on with the admin credentials (enter “admin” in both the username and password fields). You should see JATOS’ welcome screen.

In the home screen, you will find a link to create new users. If you wish, you can create a password-protected user, then logout from the admin account and log back in with your newly created credentials. You can also change the password for the admin account.

#### Step 1.2 (optional) Download and install JATOS on a server

If you want to make your experiments accessible to workers that are not sitting directly in front of your computer, you will need to install JATOS on a server. To do this, you will naturally need a configured webserver. Configuring a webserver from scratch is beyond the purposes of this tutorial, as it includes setting up regular automatic updates and automatically restarting JATOS after a reboot or crash. If you have no experience with servers, you should ask for assistance from somebody who does. If you do have experience in setting up a server, then follow the server installation instructions in the wiki pages. Briefly, it involves the same steps required for the local instance (downloading, unzipping and running the loader script), plus the additional configuration of the loader script to specify the IP address and port according to your server’s settings.

There are two other options to make JATOS accessible via the Internet. The simplest way is to install JATOS on a cloud service. The strong caveat here is that you would lose JATOS’ main advantage: ensuring data privacy. Cloud installation instructions are described in the wiki pages. A second alternative is to transform your local computer into a server, in which case a ‘local’ JATOS instance would in fact be accessible from the outside and effectively be a server instance. Again, this is beyond the scope of this tutorial, and requires some knowledge of Internet technologies and security. Multiple alternatives and tutorials are available online.

### 2. Get started: import and modify example studies

#### Step 2.1: Download an example study

If you are not proficient in JavaScript, we recommend that you modify one of the existing study scripts, rather than starting from scratch to write your own. We provide several different example studies. To get started, download the Basic Example Study from the GitHub Wiki page (https://github.com/JATOS/JATOS/wiki/Example-Studies). Save the zipped file you just downloaded to a convenient location, and do not unzip it.

#### Step 2.2: Import the Basic Example Study into your local JATOS instance

To import a study to your JATOS instance, go to JATOS in your browser and click on “Import Study” link in the header. Simply navigate to and select the.zip file with the example study that you just downloaded. (See next step if you encounter any problems at this stage).

#### Step 2.3: Identify the local JATOS folder

Use your system’s file manager to navigate to JATOS’ folder. This will help you recognize where your study’s files should be stored, and how they should be organized. As an example, if you are using Mac OS X and installed JATOS in your Applications folder, navigate to the folder /Applications/jatos-x (where x specifies your version). You should find a subfolder called “study_assets_root”, that will contain one subfolder for each of your studies. This subfolder is called study assets folder. All your scripts and other assets for any given study must be located inside its corresponding study assets folder.

To prevent overwriting, JATOS will not allow you to import a study if its corresponding folder name matches that of an existing study. The only exception to this rule is in cases where this study has the same Universally Unique Identifier (UUID). Like a study ID, a UUID is given to a study at its initial creation and is a way to identify it. Unlike a study ID, a UUID is “world-wide” unique to all JATOS instances and thus used for import/export of studies and components. If you import a study with the same UUID as an existing one, a dialog box will appear asking whether you want to overwrite all assets and/or all study’s properties. This happens if, for example, you exported a study from the server, imported it on a local instance, worked on it locally and then re-imported it back to the server.

#### Step 2.4: Run the example study

The Basic Example Study implements many of JATOS’ features, and is a good starting point on which to explore the flexibility of the study flow. The example study is self-explanatory, so the best way to understand it is to run it through.

One of JATOS’ most powerful features is its organization in components. It is not necessary to split a study into multiple components, but we recommend that you do. Having individual components makes it easier to recycle these components for new studies, such as a consent form, instructions, etc. It also naturally leads to shorter and more manageable JavaScripts.

You can display the entire study by clicking on the “Run” button on the study’s toolbar. If you want to display any component individually, click the corresponding “Run” buttons on the component’s toolbar.

#### Step 2.5: Explore and export the result data

After running the basic example, you will see that some result data were created. Navigate to the study page again, and click on “Results” on the study’s toolbar. You will see the metadata (including worker type, component start time, whether the experiment was completed, etc.) and the result data. There will be metadata associated with all components, but there will be result data only for those components that generate it explicitly. For example, the component called “Show task and submit result data” generates result data but the “Hello World” component does not. It therefore shows the word “none” displayed in its result data section.

We recommend JSON as the preferred output data format. There are multiple reasons for this. First, JSON is JavaScript’s native object notation. Second, it is already used for every study’s and component’s JSON input. Third, the JSON format is less prone to formatting errors. For example, while a missing data point in a.csv file might alter an entire line and lead to data confusion, this is not the case with the JSON format, in which each data point is accompanied by its label. Fourth, it is human-readable: it is easy to read in every text editor. Finally, multiple JSON parsers and converters are freely available.

To export your result data, simply click and select the line corresponding to the results you want to export. Click on “Export data”. This will allow you to save a text file that you can then read with a your text editor or data analysis program of choice.

#### Step 2.6: Modify the JSON input

Each component’s toolbar includes a “Properties” button that leads you to the component’s properties page. Click this button and you will see, amongst other fields, a text box containing the JSON input. These can be read by the component’s script. You can thus change the contents of the JSON input to change what is displayed in the browser. Keep in mind that text is not the only thing that can be modified via the JSON input. Paths to the experimental stimuli, timing parameters, number of trials, etc. can all be easily changed without having to hard-code them into JavaScript.

Keep in mind that each component’s JSON input can only be read, but not edited by the component’s script. Also, it cannot be shared between different components. If you want to share data between components, you could use the object jatos.studySessionData, which can be read and edited from within any of the scripts of a study. This is our preferred way to share information between components. Alternatively, you could pass parameters through the URL’s query string.

#### Step 2.7: Modify the study flow

Having multiple components in a study generates a great deal of flexibility in the study flow. The easiest way to modify a study is to change the relative position of the components within it, dragging the component to its new position. Another way to modify the study flow is by inactivating selected components. You can use the checkboxes to the left of each component to toggle them between the active and inactive states. Run the study again to confirm that inactive components are simply ignored.

JATOS can also loop, skip, or jump back and forth between components. For example, the first component might display study instructions, and the second one could ask participants a question to verify whether they understood the instructions. Depending on the participant’s response, JATOS can jump back to the instructions component (if the response was incorrect) or follow the linear order of components (if the response was correct). jatos.js includes different implementations for directing to components: by position or by component ID. The component called “Jump to Component” shows an example use of the former method.

#### Step 2.8: Examine the JavaScript files

Find the HTML file associated with the component called “Show JSON input”. If you go to this component’s properties, you will see that the filename of the HTML file (specified in the “HTML file path” field) is show_json_input.html. This file is located in the study’s subfolder, within the study assets folder. Open this file with your text editor of choice, and you will see that it contains an HTML section on top, and a JavaScript section below. Describing HTML and JavaScript coding is beyond the purpose of this tutorial (you can find plenty of resources online) but we call your attention to some specific aspects.

The header HTML section specifies a general library, namely jQuery (http://jquery.com/). We used jQuery in the Example study because it provides some useful JavaScript shortcuts. The Basic Example Study contains these plugins within its “libs” (for “libraries”) folder, so you do not need to download them separately.

The HTML header also specifies the JATOS library jatos.js. This library includes a number of useful functions to communicate with the JATOS server and submit data, retrieve the JSON input, navigate between components, etc. A complete and up-to-date list of jatos.js commands and example implementations is available in the Wiki page. All variables or functions of jatos.js start with jatos., for example the function jatos.submitResultData(resultData) lets you submit result data, or, if you need the study ID, you can get it via the variable jatos.studyId.

#### Step 2.9: Create, clone, delete and export studies or components

Managing studies and components in JATOS is intuitive. To create a new study from scratch, click on the link on top of the JATOS page, fill in the properties (some fields can be left blank) and click on “Submit”. Use the study’s toolbar to add components to any study: go to “Components” → “New”. You can also import individual components previously exported from other studies. The study and components’ toolbars contain one “More” button each. Here you will find links to export, clone or delete studies or components. Cloning (or exporting) duplicates all assets and properties but does not clone (or export) the result data. It is also worth mentioning that JATOS generates new IDs for each component if a study is cloned or exported. Therefore, if you export a study containing scripts that use fixed component IDs (e.g., with the jatos.js function jatos.startComponent(componentId)), you will need to update the value of the componentId variables after you have imported it in a different JATOS instance. Alternatively, use the position instead of the ID to index components whenever you can. Deleting a component deletes all properties and associated component results, but does not delete its assets. In contrast, deleting an entire study deletes the entire study assets folder. So be careful when you delete a study, and be sure to keep backups!

### 3. Move your local data to a server

#### Step 3.1: Set up a server

See Step 1.2 in this Tutorial and the Wiki pages for instructions for how to install JATOS on a server.

#### Step 3.2: Transfer your study from the local to the server instance

As we mentioned in the beginning of this Tutorial, it is a good idea to write and prepare your studies locally first. Transferring studies from the local to the server instance is easy: export them from your local instance, save them in a local folder, and import them directly into your server instance.

#### Step 3.3: Generate links to access a study from outside JATOS

When you are ready to collect experimental data for a study, we recommend that you set the study to the ‘Locked’ state, by clicking on the button on the study’s toolbar. JATOS will not allow any changes to a locked study’s (or its components’) properties. You can now generate links to allow others to access the study from outside JATOS. If you prefer to pair your study with AMT via an external HIT, see the next step. Go to the “More” button in the study’s toolbar, choose the link that you want to generate and distribute the link. Remember to allow your workers to access the study by checking the appropriate checkboxes in the study’s properties.

#### Step 3.4: Connect to AMT

Connecting your JATOS study to the AMT is easy, although a fair amount of clicking is required. You will need a “requester” AMT account and your study running on a JATOS server instance. In the future, we will incorporate functions to do this automatically. For now, follow the short tutorial in the Wiki pages (https://github.com/JATOS/JATOS/wiki/Connect-to-Mechanical-Turk).

## Conclusion

Because JATOS is free, GUI-based and very easy to install, it is accessible to researchers that wish to host their own custom-made experiments in their own servers. It is under continuous development, and new features will be added based on the users’ needs. We hope that this tool will prove useful to researchers from different disciplines.

## Glossary

In this glossary we clarify some of the common terms that we use in this article to refer to JATOS and its elements.

### Assets

Assets are a set of files, stored in a server, necessary to correctly display a webpage on a browser. The name “assets” can refer to many different file types, including HTML files, their associated (or embedded) JavaScripts, CSS files for formatting, images, videos, text files, audio, and libraries (e.g., the widely used jQuery and jQuery UI libraries). All assets belonging to a single study, called **study assets**, are stored within a folder, called **study assets folder**. Each study assets folder is a subfolder of the **assets root folder** located within JATOS’ folder with the name “study_assets_root”. The name and location of the assets root folder can be changed in the configuration. All components of a study share the same study assets folder.

### Client-side and server-side

An online study page, like every webpage, requires two “sides”: the client-side and the server-side. The **client-side** interprets and displays an HTML file (often together with JavaScript) in an Internet browser. The **server-side** stores all the assets (e.g., HTML and JavaScript files, images, videos, audio and text files, etc.) that the client-side will request. In addition, JATOS’ server is paired with a database that can store each participants’ responses. While study participants can, in principle, read and modify everything in the client-side, the server-side is secure, as it can be protected from general access.

### Component

A JATOS component is a part of a study, associated with a webpage (specified by an HTML file) and properties. For example, a component could display the study instructions, react to a key press and record how long the instructions were presented on the screen.

### Database

JATOS is paired with a database that stores, among other things, all participants’ results, including both the **result data** and **metadata**.

### JSON format

**JSON** stands for JavaScript Object Notation and is a simple, human-readable, and robust way to exchange data with JavaScripts. JSON is flexible, in that it admits different data types, including numbers, strings, booleans and arrays.

### JSON input

The **JSON input** is a JSON string, specified in the component’s or the study’s properties. Any given component can access its own and the study’s JSON input, but it cannot access that from another component. The JSON input might specify, for example, the text to be displayed, the number of trials for each condition, the response alternatives or the maximum allowed response time window. The JSON input is static and cannot be changed by e.g. the participant’s responses (see **study session data**for a way to share editable JSON data between different components of the same study).

### jatos.js

jatos.js is a JavaScript library that wraps JATOS’ public API calls (operations that the client side can send as requests to the server), that are used within component's scripts. A full list of functions and variables is available in the JATOS Wiki.

### Metadata

Metadata are data that are not explicitly sent to the server from within each component’s JavaScript, but that JATOS associates automatically with each data entry. The metadata include **worker type** and ID, component start time and duration and whether the component was completed.

### Result data

Result data in JATOS simply refers to the data that was sent to the server from each component’s JavaScript. These data are stored in the **database** after a participant completed a component, together with the **metadata**.

### Study

A study in JATOS is a group of components together with a series of properties. Study-specific properties (*i*.*e*., properties that apply to the entire study but not to the individual components directly) are set in the **study’s properties**. They include a description of the study, the **worker types** that are allowed to access the study, the **study’s JSON input**, the study’s folder name, etc.

### Study’s and component’s toolbars

Studies and components can be cloned, deleted, exported, shown on the browser, etc. Two different toolbars contain buttons that provide links to these functions. The **study’s toolbar**, located in the top of JATOS’ study page, includes study-specific links. Each **component’s toolbar** is a group of buttons on the right of JATOS’ study page, associated with a single component and includes component-specific links (see Fig 1).

### Study and component results

Each component run generates its own output data. These, combined with the component’s metadata, make up the **component result**. All component results of a whole study run plus the study’s metadata make up the **study result**.

### Study session data

The session data can be accessed and modified by every component of a study. This is therefore a convenient way to share data between different components. The session data are deleted once a study is finished.

### Users and members

We refer to researchers using JATOS as **users**. Multiple users can access and host their studies on a single JATOS instance. Each study is associated with a list of **members**, who have editing privileges to it.

### Worker types

Following AMT’s terminology, a worker in JATOS is a person who runs some or all of the components of a study. This includes not only study participants (external workers) but also the researchers setting up the study, if they run the study (or any of its components) internally from within JATOS. Different worker types access a study in different ways. For example, some workers can access the same study multiple times, whereas others can do it only once. See the Wiki pages for details on worker types.
