# Correction: “Just Another Tool for Online Studies” (JATOS): An Easy Solution for Setup and Management of Web Servers Supporting Online Studies

**DOI:** 10.1371/journal.pone.0134073

**Published:** 2015-07-21

**Authors:** Kristian Lange, Simone Kühn, Elisa Filevich

There is an error in the affiliation for the first author. Kristian Lange is not affiliated with #1, and her affiliation should be: Independent Researcher, Berlin, Germany.
